# Supplementary material for: A UMLS-based spell checker for natural language processing in vaccine safety
Source: BMC Med Inform Decis Mak. 2007 Feb 12;7:3. doi: 10.1186/1472-6947-7-3 (PMC1805499; doi:10.1186/1472-6947-7-3)
Supplement: Additional file 13 — RAP application directory. Sets up directory for RAP (RDF application for PHP) source code [file 1472-6947-7-3-S13.gz › rap/api/util/adodb/tests/client.php]

php
/\*\*
\* V4.50 6 July 2004 (c) 2001-2002 John Lim (jlim@natsoft.com.my). All rights reserved.
\* Released under both BSD license and Lesser GPL library license.
Whenever there is any discrepancy between the two licenses,
the BSD license will take precedence.
\*
\* set tabs to 8
\*/
// documentation on usage is at http://php.weblogs.com/adodb\_csv
include('../adodb.inc.php');
include('../tohtml.inc.php');
function &send2server($url,$sql)
{
$url .= '?sql='.urlencode($sql);
print "<p$url";
$rs = csv2rs($url,$err);
if ($err) print $err;
return $rs;
}
function print\_pre($s)
{
print "

```
";print_r($s);print "
```

";
}
$serverURL = 'http://localhost/php/phplens/adodb/server.php';
$testhttp = false;
$sql1 = "insertz into products (productname) values ('testprod 1')";
$sql2 = "insert into products (productname) values ('testprod 1')";
$sql3 = "insert into products (productname) values ('testprod 2')";
$sql4 = "delete from products where productid>80";
$sql5 = 'select \* from products';
if ($testhttp) {
print "Client Driver Tests

";
print "

### Test Error

";
$rs = send2server($serverURL,$sql1);
print\_pre($rs);
print "

---

";
print "

### Test Insert

";
$rs = send2server($serverURL,$sql2);
print\_pre($rs);
print "

---

";
print "

### Test Insert2

";
$rs = send2server($serverURL,$sql3);
print\_pre($rs);
print "

---

";
print "

### Test Delete

";
$rs = send2server($serverURL,$sql4);
print\_pre($rs);
print "

---

";
print "

### Test Select

";
$rs = send2server($serverURL,$sql5);
if ($rs) rs2html($rs);
print "

---

";
}
print "

# CLIENT Driver Tests

";
$conn = ADONewConnection('csv');
$conn->Connect($serverURL);
$conn->debug = true;
print "

### Bad SQL

";
$rs = $conn->Execute($sql1);
print "

### Insert SQL 1

";
$rs = $conn->Execute($sql2);
print "

### Insert SQL 2

";
$rs = $conn->Execute($sql3);
print "

### Select SQL

";
$rs = $conn->Execute($sql5);
if ($rs) rs2html($rs);
print "

### Delete SQL

";
$rs = $conn->Execute($sql4);
print "

### Select SQL

";
$rs = $conn->Execute($sql5);
if ($rs) rs2html($rs);
/\* EXPECTED RESULTS FOR HTTP TEST:
Test Insert
http://localhost/php/adodb/server.php?sql=insert+into+products+%28productname%29+values+%28%27testprod%27%29
adorecordset Object
(
[dataProvider] => native
[fields] =>
[blobSize] => 64
[canSeek] =>
[EOF] => 1
[emptyTimeStamp] =>
[emptyDate] =>
[debug] =>
[timeToLive] => 0
[bind] =>
[\_numOfRows] => -1
[\_numOfFields] => 0
[\_queryID] => 1
[\_currentRow] => -1
[\_closed] =>
[\_inited] =>
[sql] => insert into products (productname) values ('testprod')
[affectedrows] => 1
[insertid] => 81
)
--------------------------------------------------------------------------------
Test Insert2
http://localhost/php/adodb/server.php?sql=insert+into+products+%28productname%29+values+%28%27testprod%27%29
adorecordset Object
(
[dataProvider] => native
[fields] =>
[blobSize] => 64
[canSeek] =>
[EOF] => 1
[emptyTimeStamp] =>
[emptyDate] =>
[debug] =>
[timeToLive] => 0
[bind] =>
[\_numOfRows] => -1
[\_numOfFields] => 0
[\_queryID] => 1
[\_currentRow] => -1
[\_closed] =>
[\_inited] =>
[sql] => insert into products (productname) values ('testprod')
[affectedrows] => 1
[insertid] => 82
)
--------------------------------------------------------------------------------
Test Delete
http://localhost/php/adodb/server.php?sql=delete+from+products+where+productid%3E80
adorecordset Object
(
[dataProvider] => native
[fields] =>
[blobSize] => 64
[canSeek] =>
[EOF] => 1
[emptyTimeStamp] =>
[emptyDate] =>
[debug] =>
[timeToLive] => 0
[bind] =>
[\_numOfRows] => -1
[\_numOfFields] => 0
[\_queryID] => 1
[\_currentRow] => -1
[\_closed] =>
[\_inited] =>
[sql] => delete from products where productid>80
[affectedrows] => 2
[insertid] => 0
)
[more stuff deleted]
.
.
.
\*/
?>
